# Physico-Mechanical and Hygro-Thermal Properties of Compressed Earth Blocks Stabilized with Industrial and Agro By-Product Binders

**DOI:** 10.3390/ma13173769

**Published:** 2020-08-26

**Authors:** Philbert Nshimiyimana, Adamah Messan, Luc Courard

**Affiliations:** 1 Laboratoire Eco-Matériaux et Habitats Durables (LEMHaD), Institut International d’Ingénierie de l’Eau et de l’Environnement (Institut 2iE), Rue de la Science, Ouagadougou 01 BP 594, Burkina Faso; adamah.messan@2ie-edu.org; 2 Department of Urban and Environmental Engineering (UEE), Université de Liège, Allée de la Découverte 9, 4000 Liège, Belgium; luc.courard@uliege.be

**Keywords:** bulk density, compressed earth block, compressive strength, hygrothermal property, byproduct binder, structural efficiency, thermal efficiency

## Abstract

This study investigated the engineering properties of compressed earth blocks (CEBs) stabilized with by-product binders: calcium carbide residue (CCR) and rice husk ash (RHA). The dry mixtures were prepared using the earthen material and 0–25 wt% CCR, firstly, and 20 wt% CCR partially substituted by the RHA (CCR:RHA in 20:0–12:8 ratios), secondly. The appropriate amount of water was thoroughly mixed with the dry mixtures. The moistened mixtures were manually compressed into CEBs, cured, dried, and tested. The stabilization of CEBs with CCR increased the dry compressive strength (CS) from 1.1 MPa with 0% CCR to 4.3 MPa with 10% CCR and above; decreased the bulk density (ρ_b_: 1800–1475 kg/m^3^) and increased the total porosity (TP:35–45%). This resulted in the improvement of the coefficient of structural efficiency (CSE: 610–3050 Pa∙m^3^/kg). It also improved the thermal efficiency given the decrease of the thermal conductivity (λ: 1.02–0.69 W/m∙K), thermal diffusivity (a: 6.3 × 10^−7^ to 4.7 × 10^−7^ m^2^/s) and thermal penetration depth (δp: 0.13–0.11 m). The RHA further improved the CS up to 7 MPa, reaching the optimum with 16:4 CCR:RHA (ρ_b_: 1575 kg/m^3^ and TP: 40%). The latter reached higher CSE (4460 Pa∙m^3^/kg) than cement stabilized CEBs (3540 Pa∙m^3^/kg). It reached lower λ (0.64 w/m∙K), a (4.1 × 10^−7^ m^2^/s) and δp (0.11 m) than cement CEBs (1.01 w/m∙K, 6.8 × 10^−7^ m^2^/s, and 0.14 m). Additionally, the stabilization of CEBs with by-products improved the moisture sorption capacity. The improvement of the structural and thermal efficiency of CEBs by the stabilization with by-product binders is beneficial for load-bearing capacity and thermal performances in multi-storey buildings.

## 1. Introduction

Despite its benefits on mechanical and durability properties, the stabilization of raw earthen material using cement is criticized for tempering with its natural advantages; i.e., low energy and carbon footprint, recyclability, moisture exchange capacity, and hygrothermal performances, among others [1,2,3]. Alternatively, the stabilization of raw earth can be achieved by mechanical compaction using relatively low to high pressure (2–10 MPa) [4], or hypercompaction up to 100 MPa [5]. Nevertheless, the hypercompaction requires high-end equipment and energy to reach such compaction pressure. Therefore, the stabilization attempts were also achieved by physical incorporation of granular particles such as sand and aggregates/fibers in the earthen material prior to compaction [6,7].

The compaction and/or incorporation of granulates or fibers can affect the mechanical and/or thermal performances due to their physico-mechanical effects in the earthen matrix. However, this approach does not necessarily guarantee the earthen material to resist in contact with liquid water [8], mainly for the earth which has low inner cohesion. However, it opens up the opportunity of the addition of by-product binders from agriculture, industrial, or municipal wastes in the earthen construction material, in general, and compressed earth blocks (CEBs), in particular. This is beneficial in terms of reusing the wastes which would otherwise be hazardous to the environment and possibly keeping the earthen material at low embodied energy and recyclable. Thus, the stabilization of earth was carried out by substitution of the cement and lime with by-product binders for improving the performances and sustainability of the raw earth [9].

The so called “supplementary cementitious materials (SCM) like silica fume, fly ash, ground granulated blast furnace slag or other pozzolans are the most frequently used stabilizers, either alone or in association” [10], contain a good amount of reactive silica or silicoalumina, and the soil intervenes in a time- and temperature-dependent pozzolanic reaction with the interstitial lime-rich water [10,11,12]. Additionally, a lime-rich industrial by-product of calcium carbide residue (CCR), on its own or combined with pozzolanic materials, can substitute the lime and be used for the stabilization of CEBs [13]. This results in the development of cementitious hydrated products, similarly to those from the hydration of cement, responsible for the development of the properties of CEBs [10,11,12,13,14,15].

Some studies have previously attempted to stabilize the CEBs using by-product binders. Muntohar [14] stabilized the CEBs using rice husk ash (RHA), a pozzolan from the calcination of a by-product from rice paddies, in partial substitution of lime. It improved the compressive strength and decreased water absorption of CEBs. Masuka et al. [15] used coal fly ash, lime, and wood aggregates for the production of earth blocks. The authors reported comparable or even higher dry compressive strength than cement stabilized blocks, but deplored the necessity of little (4%) cement content for better wet strength. On the other hand, Izemmouren et al. [12] assessed the stabilization effect of the combination of lime and natural pozzolana and steam-curing up to 75 °C on the performances of CEBs. The steam curing accelerated the pozzolanic reaction to reach the optimum compressive strength in 24 h, while the natural pozzolana improved other mechanical and durability properties of CEBs.

A recent study [11] showed that the mixtures of earthen material with CCR or earthen material with CCR:RHA, cured in ambient condition (30 ± 5 °C), undergo the chemico-microstructural changes throughout the pozzolanic reaction. These changes considerably improved the compressive strength of CEBs stabilized with up to 8% CCR, beyond which it sharply decreased due to the lack of effective interaction with the earthen materials [13]. By contrast, the strength continuously increased up to 20% CCR for CEBs cured at elevated controlled temperature of 40 °C [16].

The present study assesses “how the stabilization using by-product binders affect the macro-engineering properties of CEBs for structural applications?” compared to unstabilized and cement-stabilized CEBs. Unlike the previous studies [13,16], this study took into account the recommendations [17] on the water demand of the CCR for the production of stabilized CEBs and curing at ambient temperature in a lab (30 ± 5 °C). It specifically investigates the effect of stabilization with the CCR and CCR:RHA on the compressive strength of CEBs and other engineering properties; such as bulk density, hygrothermal properties, and structural and thermal efficiency for sustainable applications in modern building constructions.

## 2. Materials and Methods

### 2.1. Materials

A kaolinite-rich earthen material was stabilized with calcium carbide residue (CCR) and rice husk ash, available in the vicinity of Ouagadougou, Burkina Faso, for the production of CEBs. The physico-chemical and mineral characterizations of the materials were reported in previous studies [13,16]. The earthen material (Kamboinse, Burkina Faso) is a silt-clay of medium to high plasticity (average plasticity index: 20 and average liquidity limit: 50), containing 20% clay particles (<2 µm), and 55% kaolinite, 20% quartz [16]. It has a specific density of 2.75 [13]. In the previous study, the kaolinite-rich earthen material had better pozzolanic reactivity with the CCR than the quartz-rich material [17]. The undersize of representative earthen materials was dry sieved on 5 mm for the production of stabilized CEBs.

The CCR is finer than 125 µm, after grinding and sieving. It has a median diameter D_50_ of 20.5 µm, a specific density of 2.49, and Blaine and BET surface area of 8286 cm^2^/g and 14 m^2^/g, respectively. It contains up to 40% hydrated lime Ca(OH)_2_ and carbonates [13]. The RHA was produced by calcination of the rice husk in optimum conditions (500 °C for 2 h). The RHA was ground and sieved on 80 µm to reach D_50_ of 11 µm, with a specific density of 2.25, and Blaine and BET surface area of 26,114 cm^2^/g and 154 m^2^/g, respectively. According to the test proposed by Mehta [18], the RHA is mainly amorphous with the reactive (amorphous) fraction of 89%.

### 2.2. Design, Production and Curing of Stabilized CEBs

Some mixtures were prepared using the earthen material and 0–25 wt% CCR alone. Other mixtures were prepared using the earthen material and 20 wt% CCR partially substituted with the RHA (i.e., CCR:RHA in 20:0–12:8 ratios). Moreover, control mixtures were produced using the earthen material and 8% cement (8CEM). The mixtures were thoroughly dry-mixed until apparent homogeneity. The appropriate moisture content was added to the dry mixtures and mixed until homogeneous moisture distribution. The optimum moisture content (OMC) was determined by the static compaction method, according to CDE [19], and used for the mixtures containing the CCR, contrary to the previous study [13]. The OMC (%) for achieving maximum dry density of the mixtures linearly increased with CCR content (%), i.e., OMC = 0.21 × CCR + 17. The moisture content of 22% was used for the mixtures containing CCR:RHA.

An appropriate amount of moistened mixtures was manually compressed in a prismatic mold (295 mm × 140 mm × 95 mm as recommended by XP P13-901 standard [20]) of a terstaram machine (Appro-Techno sprl, Couvin, Belgium) to produce CEBs. The terstaram machine was designed to offer a compaction pressure of about 35 bars [21]. The stabilized CEBs were wrapped in plastic bags and cured at constant moisture of production for 45 days at ambient temperature in a laboratory (30 ± 5 °C), as suggested by the previous study [17]. Cured CEBs were dried at 40 ± 2 °C until the change of mass is less than 0.1% between 24 h, before testing their properties.

### 2.3. Characterization of Stabilized CEBs

#### 2.3.1. Physical and Mechanical Proprieties

The characterizations were carried out on at least three specimens of CEBs for the consideration of average and standard deviation values. The bulk density, ρ_b_ (kg/m^3^), of dry CEBs of mass, Md (kg) was determined using Equation (1) after hydraulic weighing [22]. Msat.wt (kg) and Msat.air (kg) are mass of water saturated CEBs after 24 h of immersion respectively weighed in water and in air and ρ_wt_ (1000 kg/m^3^) is the density of water. The total porosity, TP (%), was estimated form the ratio of the bulk density, ρ_b_, of CEBs and the equivalent specific density, ρ_s,_ of constituting particles using Equation (2). The compressive strength was tested on the stack of two halves of CEBs, in dry and wet conditions after immersion in water for 2 h, using hydraulic press (Proeti safr, Madrid, Spain) equipped with a 300 kN capacity load cell at loading rate of 0.2 mm/s, referring to XP P13-901 standard [20]. The compressive strength, Rc (MPa), was calculated using Equation (3), where Fr (kN) is the maximum load at failure and S (cm^2^) is the area of applied surface:

(1) $$\rho_{b}=\mathrm{Md}\;\times \;\rho_{\mathrm{wt}}/\left(\mathrm{Msat}.\mathrm{air}-\mathrm{Msat}.\mathrm{wt}\right)$$

(2) $$\mathrm{TP}=100\;\times \;\left(1-\rho_{b}\right)/\rho_{s}$$

(3) $$R_{c}=10\;\times \;F_{r}/S$$

#### 2.3.2. Hygrothermal Proprieties

The thermal properties were measured on dry samples and wet samples containing different amount of water. The thermal effusivity, E (J/m^2^·K s^1/2^), was measured on specimens of size 6 cm × 4 cm × 3 cm, whose thickness (3 cm) does not allow the thermal flux to cross through the specimen and the volumetric thermal capacity, Cp (J/m^3^·K) on size 6 cm × 4 cm × 1 cm which allows the flux to cross through the specimen [21,23]. The specific heat capacity (J/kg∙K) was determined from the ratio of the volumetric thermal capacity, Cp (J/m^3^·K) and the bulk density, ρ_b_ (kg/m^3^) of CEBs. The thermal conductivity, λ (W/m∙K), thermal diffusivity, a (m^2^/s), and thermal penetration depth, δ_p_ (m), were respectively determined using Equations (4)–(6) over a period, T (s), of 24 h (86,400 s).

(4) $$\lambda =E^{2}/\left(\mathrm{Cp}\right),$$

(5) $$a=\lambda /\left(\mathrm{Cp}\right),$$

(6) $$\delta_{p}\;=\;\sqrt{\left(a\;\times \;T/\pi \right)},$$

Sorption capacity was measured on CEB specimens (6 cm × 4 cm × 1 cm) using the method of saturated salt solutions, according to the standard EN ISO 12571 [24]. The specimens were dried at 40 ± 2 °C, at relative humidity (Ψ: 3% in presence of silica gel). They were exposed to continuously increasing relative humidity (Ψ: 9, 33, 43, 69, 75, 83, 97%) produced in the desiccator by different saturated salt solutions (KOH, MgCl.6H_2_O, K_2_CO_3_, KI, NaCl, KCl, K_2_Cr_2_O_4_) conditioned at 20 ± 2 °C. The equilibrium moisture content, EMC (%), adsorbed by the specimens in equilibrium with the relative humidity in the desiccator (2–3 weeks) was determined between the dry mass, Md, and wet mass, Mw, respectively before and after exposure to each Ψ (Equation (7)). The isotherms of the EMC were fitted using the Guggenheim-Anderson-de Boer (GAB) model (Equation (8)). This model was previously used to cover large interval of Ψ (5–95%) for CEBs and other construction materials [25].

(7) $$\mathrm{EMC}=100\times \left(\mathrm{Mw}-\mathrm{Md}\right)/\mathrm{Md},$$

(8) $$\mathrm{EMC}=\frac{C\;\times \;k\;\times \;w_{m}}{\left(1-k\;\times \;\Psi \right)x\left(1-k\;\times \;\Psi +C\;\times \;k\;\times \;\Psi \right)}x\Psi ,$$

## 3. Results and Discussion

### 3.1. Physical and Mechanical Properties of Stabilized CEBs

**Figure 1:**
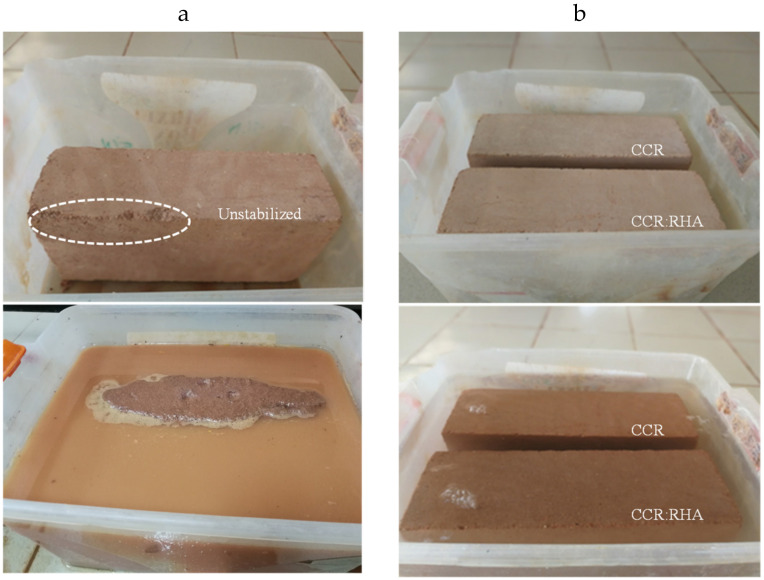
Apparent behaviors of CEBs in dry state (top) versus immersed in water for two h (bottom): (**a**) unstabilized; (**b**) stabilized with CCR or CCR:RHA.

The apparent behavior and bulk density, as well as mechanical resistance of CEBs were improved by the stabilization with by-product binders. Unstabilized CEBs were friable in dry state (degraded corners) and completely degraded in contact with liquid water (Figure 1a). However, the CEBs stabilized with the CCR or CCR:RHA were very stable (sharp corners) both in dry and contact with liquid water (Figure 1b).

#### 3.1.1. Bulk Density and Total Porosity

**Figure 2:**
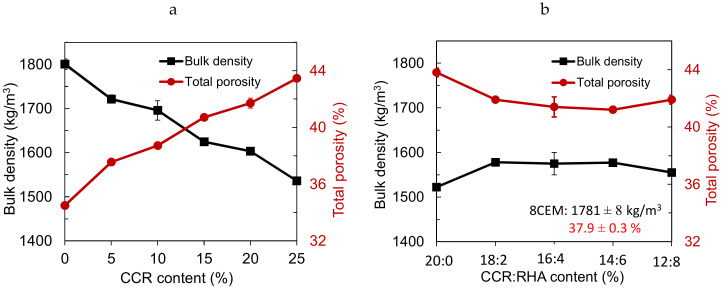
Bulk density and total porosity of CEBs stabilized with (**a**) CCR, (**b**) CCR:RHA.

The addition of 0–25% CCR decreased the bulk density of CEB in the range of 1800 to 1477 kg/m^3^, following the increase of the total porosity in the range of 35–45% (Figure 2a). The increase of the total porosity with the addition of CCR can be related to the OMC for the production of stabilized CEBs which increased in the range of 17–23%. By contrast, the partial substitution of 20% CCR by the RHA (CCR:RHA) slightly increased the bulk density from 1522 kg/m^3^ (20:0% CCR:RHA, i.e., 20% CCR alone) to 1578 kg/m^3^ (18:2% CCR:RHA). This respectively corresponds to the decrease of the total porosity from 44 to 41%. Beyond 18:2% CCR:RHA, the bulk density and total porosity tend to be constant (Figure 2b).

It is noteworthy to remind that the production moisture (22%) for CEBs stabilized with CCR:RHA (various ratios) was the same as CEBs stabilized with 20% CCR alone. The tendency of the bulk density and total porosity to reach the constant values suggests that, at the similar content of production moisture, the CCR:RHA can produce denser and lesser porous CEBs than the CCR alone. This suggests that the bulk density and total porosity can further be improved by optimization of the moisture of production of CEBs stabilized with CCR:RHA. The bulk density and the porosity of CEBs stabilized with by-product binders are respectively lower than 1780 kg/m^3^ and higher than 36% for CEBs stabilized with 8% cement. Furthermore, Table 1 and Table 2 summarize the averages and coefficient of variation (CV) of the bulk density and total porosity of CEBs stabilized with the by-products; it shows a similar range of variations (CV < 2).

In the present study, the bulk density and total porosity of unstabilized CEBs or stabilized with by-product binders are respectively lower than 2000 kg/m^3^ and higher than 25% reported in the literature for unstabilized CEBs produced using normal compaction pressure (<2 MPa) [26]. This can be related to the type of material used in the present study which required an OMC (>17%), far higher than 13% used in the literature [26]. Similar observation was made for the CEBs stabilized with 8% cement in the present study which have bulk density (1780 kg/m^3^) and porosity (36%) respectively lower and higher than their counterpart in the literature, i.e., 1810 kg/m^3^ and 30% [27]. The latter was produced using OMC of 9.5% and compaction pressure of about 3.6 MPa [27].

By contrast, the values of the bulk density and total porosity of CEBs stabilized with 8% cement are respectively higher and lower than the values for CEBs stabilized with CCR or CCR:RHA, in the present study. Indeed, it was previously reported that the bulk density decreases with increasing content of lime (CCR in the present study), while it increases with cement stabilization [27,28]. This cannot only be related to the low demand of production moisture of CEBs stabilized with cement, but also to the higher specific density of cement (3.1) compared to that of CCR (2.49).

#### 3.1.2. Compressive Strength in Dry and Wet Conditions

**Figure 3:**
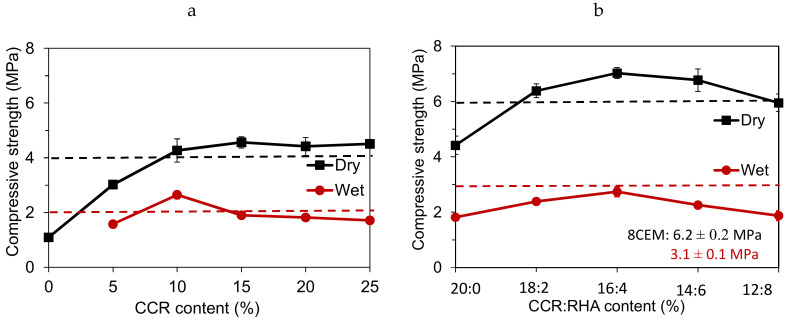
Compressive strength of CEBs stabilized with (**a**) CCR, (**b**) CCR:RHA: Comparison with 4 and 6 MPa of dry compressive strength required for load bearing walls respectively in two- and three-storey buildings [29].

Figure 3 details the evolution of the compressive strength in dry and wet conditions (dry and wet strength) of CEBs stabilized with by-product binders (CCR and RHA) cured in ambient condition of the lab (30 ± 5 °C). The average dry compressive strength significantly increased (2.8 times) by addition of CCR, from 1.2 MPa (unstabilized: 0% CCR) to 4.6 MPa (25% CCR), i.e., (4.6–1.2)/1.2. Beyond 10% CCR, the compressive strength (4.3 MPa) did not record significant improvement (Figure 3a). Additionally, the average wet compressive strength of CEBs stabilized with 5–25% CCR slightly improved, reaching the maximum value of 2.7 MPa with 10% CCR; beyond which it decreased to less than 2 MPa (Figure 3a). The wet compressive strength of unstabilized (0% CCR) CEBs could not be determined as they immediately degraded in water (Figure 1a). It can be suggested that CEBs should be stabilized with at least 10% CCR to reach the dry and wet compressive strength respectively of 4 and 2 MPa; these values are required for wall construction of two storey building [29].

The asymptotic evolution of the compressive strength (>10% CCR, Figure 2a) can be explained by ineffective pozzolanic reaction between the clay earthen material and excess CCR [13]. However, it is comparable to the compressive strength of CEBs produced using the same OMC (earth and 10–20% CCR) and cured at 40 °C (3.9–4.7 MPa), but higher than that of CEBs cured at 20 °C (2.3–2.5 MPa) [17]. This confirms the benefits of the stabilization with lime-rich binder in warm regions; where the ambient temperature (30 ± 5 °C) would be enough to activate the pozzolanic reaction between the kaolinite-rich earthen material and lime-rich binders.

For the CCR content in the range of ineffective pozzolanic reactivity (>10%), the partial substitution of the 20% CCR by the RHA (CCR:RHA) further improved the compressive strength of CEBs. The dry compressive strength reached the average maximum value of 7 MPa with 16:4% CCR:RHA (Figure 3b); it increased 0.6 times with respect to 4.4 MPa reached with 20:0% CCR:RHA, i.e., (7–4.4)/4.4. It clearly points out the usefulness of the substitution by the RHA at high content of the CCR (20%) on improving the compressive strength without compromising (increasing) the porosity of CEBs (§3.1.1). This improvement is significant in sense that it allows to produce CEBs suitable for bearing load in walls of three storey buildings, i.e., reaching the dry compressive strength of 6 MPa [29].

Nevertheless, the load-bearing capacity of CCR:RHA stabilized CEBs was compromised in wet conditions as the wet compressive strength reached the maximum value of 2.7 MPa with 16:4% CCR:RHA. It failed to reach the required strength of 3 MPa for applications in facing masonry of three-storey buildings [29]. However, these CEBs can potential be used in dry environment or subjected to surface or architectural protection against water. The CCR:RHA-stabilized CEBs achieved the optimum compressive strength which is comparable to that of CEBs stabilized with 8% cement (CEM), which recorded 6.2 and 3.1 MPa, respectively, in dry and wet conditions. This improvement of the compressive strength is mainly related to the chemical interactions and microstructural changes which were accelerated by the RHA in the mixtures of earthen material and binders (CCR:RHA) [11].

Table 1 and Table 2 further summarize the average compressive strength of stabilized CEBs along with the coefficients of variations (CV), in dry and wet conditions. It shows that the compressive strength records similar degree of variation (CV < 10%) for the CEBs stabilized with CCR and CCR:RHA. Table 1 and Table 2 also present the coefficient of water strength (CWS): ratio between the wet and dry compressive strength. The CWS evolved in the range of 0.4 and 0.6 for CEBs stabilized with by-product binders; compared to 0.5 for CEBs stabilized with 8% cement. This is indeed a good indicator of the durability of stabilized CEB, in addition to the resistance to water degradation (Figure 1b). However, further tests need to be done to assess the long-term behaviors.

The literature reported lower or comparable values of the compressive strength for CEBs stabilized with cement, lime or geopolymer. For instance, Bogas et al. [27] reported the dry compressive strength of 2.3, 3.3 and 5.5 MPa respectively for unstabilized CEBs, CEBs stabilized with 4:4% cement:lime and 8% cement. The authors [27] also recorded drastic decrease of the compressive strength of CEB in direct contact with water. Sore et al. [21] reported that CEBs stabilized with 10% geopolymer reached dry compressive strength comparable to the present study, i.e., 4.4 MPa from 1.4 MPa for unstabilized CEBs. The binder content (cement, lime) clearly affects the strength of CEBs, contrary to the porosity which is mostly affected by the compaction pressure (consolidation) and/or moisture of production [26].

This can explain the increase of the compressive strength and decrease of the bulk density of stabilized CEBs, in the present study. It is mainly due to the counteracting phenomena: (1) formation of the cementitious hydrates responsible for the binding cohesion and improvement of strength, (2) increase of the total porosity from the increase of OMC which decreases the cohesion, bulk density, and strength. The first phenomenon prevails during the improvement of the compressive strength of stabilized CEBs, i.e., 0 to 10% CCR or 20:0 to 16:4% CCR:RHA, beyond which there is a compromise of the two phenomena. Although, the tensile strength was not determined in the present study, it would be interesting to have an idea on its evolution. This can be predicted from the relationship reported in the literature, between the bending strength (Rb) and the compressive strength (Rc) of earth blocks, such that Rb = Rc/6 [30].

#### 3.1.3. Structural Efficiency

The coefficient of structural efficiency (CSE) is an important physico-mechanical parameter to assess the contribution of the strength and density of CEBs toward the load bearing capacity in building construction. The CSE was determined as the ratio between the dry compressive strength and bulk density of CEBs. The aim is to maximize the resistance (strength) at the same time minimize the weight (density) of material in order to improve the load-bearing capacity. The stabilization with CCR remarkably improved the CSE which increased more than 3 times, i.e., from 609 Pa∙m^3^/kg (J/kg) for unstabilized (0% CCR) to 2530–3050 J/kg for 10–25% CCR-stabilized CEBs (Table 1). This implies that the CEBs stabilized with higher content of CCR (25%) can bear more loads given that they are lighter than CEBs stabilized with lower content of CCR (10%), while both have comparable resistance. This suggests that the decrease of bulk density has a positive effect on the structural efficiency of stabilized CEBs.

The stabilization with CCR:RHA further increased (0.5 times) the bearing capacity of CEBs, from a CSE of 2902J/kg with 20:0% CCR:RHA to a maximum value of 4462 J/kg with 16:4% CCR:RHA (Table 2). This suggests that the CEBs stabilized with 16:4% CCR:RHA are not only the optimum design regarding the compressive strength, but also the bearing capacity. In fact, the CSE of CEBs stabilized with 16:4% CCR:RHA is even higher than the CSE (3547 J/kg) of CEBs stabilized with 8% cement. Moreover, the CSE of optimum design (16:4% CCR:RHA) is higher than CSE deduced from the literature, such as 3040 J/kg for CEBs stabilized with 8% cement [27] and 4290 J/kg with 8% cement [21]. The CSE of CEBs stabilized with 10% CCR is also higher than 2450 J/kg for CEBs stabilized with 10% geopolymer [21].

### 3.2. Hygrothermal Properties of Stabilized CEBs

#### 3.2.1. Thermal Effusivity and Specific Thermal Capacity

The thermal properties of CEBs were also improved by the stabilization with by-product binders. Specifically, the average thermal effusivity decreased in the range of 1290 to 1010 J/m^2^∙K∙s^1/2^, measured for CEBs stabilized with 0 to 25% CCR (Table 1). It further evolved in the range of 1110 to 970 J/m^2^∙K∙s^1/2^ for CEBs stabilized with CCR:RHA, reaching the minimum value with 16:4% CCR:RHA (Table 2). The values of the thermal effusivity of CEBs stabilized with CCR and CCR:RHA are lower than 1231 J/m^2^∙K∙s^1/2^ for CEBs stabilized with 8% cement.

The specific thermal capacity measured for CEBs stabilized with 0–25% CCR increased in the range of 890 to 1000 J/kg∙K (Table 1). It evolved in the range of 970 and 880 J/kg∙K for the CEBs stabilized with CCR:RHA, reaching the maximum with 20:0% CCR:RHA (Table 2). These values are higher than the thermal capacity of 844 J/kg∙K for 8% cement stabilized CEBs. Table 1 and Table 2 further show that both the thermal effusivity and capacity record the same range of variations (CV < 4) for all CEBs.

**Figure 4:**
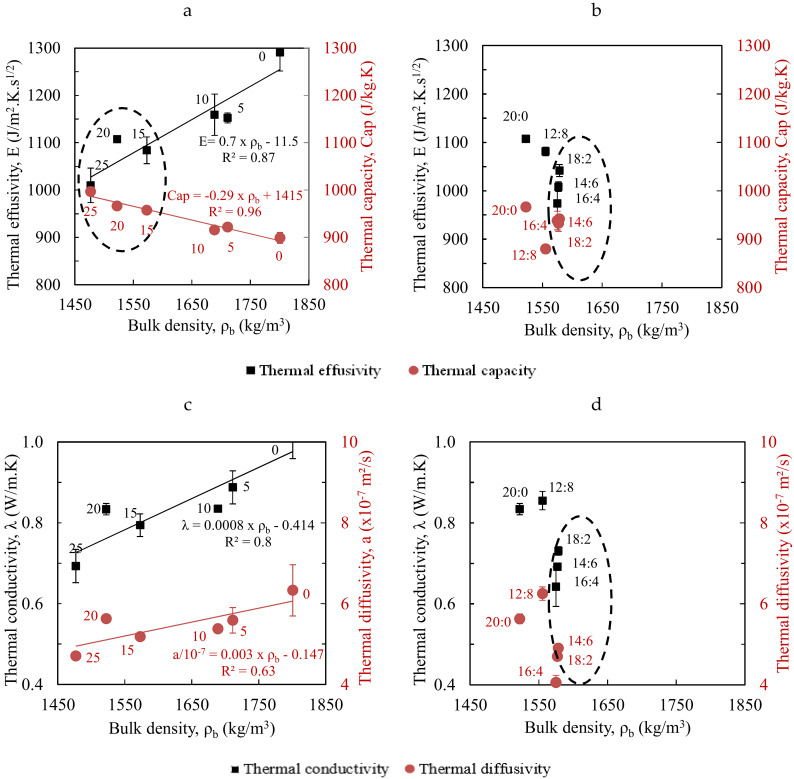
Evolution of the thermal effusivity and specific capacity of CEBs stabilized with (**a**) CCR, and (**b**) CCR:RHA, and thermal conductivity and diffusivity of CEBs stabilized with (**c**) CCR, and (**d**) CCR:RHA, with respect to the bulk density. Indices represents the content of binders.

The thermal effusivity tends to increase with the bulk density of CEB and particularly shows good correlations for CEBs stabilized with CCR (Figure 4a). Similar evolution was previously reported for geopolymer-stabilized and unstabilized CEBs [21,26]. The thermal capacity also decreases linearly with the bulk density for CCR-stabilized CEBs (Figure 4b). Ideal building materials for application in facing wall masonry in warm regions should have the lowest thermal effusivity, i.e., the lowest ability to absorb heat from the surrounding, and/or the highest specific thermal capacity, i.e., the highest capacity to store the absorbed heat [31]. This would results in low thermal conductivity and diffusivity and, thus, high thermal inertia. Figure 4 presents the possibility to respectively minimize and maximize the values (areas in the circles) of thermal effusivity and specific capacity with respect to the bulk density of CEBs. It suggests that CEBs stabilized 15–25% CCR (Figure 4a,b) or 14:6–18:2% CCR:RHA (Figure 4c,d) would be better designs for thermal applications in warm regions.

#### 3.2.2. Thermal Conductivity and Thermal Diffusivity

The average thermal conductivity decreased from 1.02 to 0.69 W/m∙K for CEBs stabilized with 0 to 25% CCR (Table 1). CEBs stabilized with CCR:RHA recorded the minimum value of the thermal conductivity of 0.64 W/m∙K with 16:4% CCR:RHA from 0.83 W/m∙K with 20:0% CCR:RHA. These values are lower than the thermal conductivity of 1.01 W/m∙K reached for CEBs stabilized with 8% cement (Table 2). The thermal diffusivity of CEBs similarly decreased from 6.3 × 10^−7^ to 4.7 × 10^−7^ m^2^/s with 0 to 25% CCR (Table 1). It reached the minimum value of 4.1 × 10^−7^m^2^/s with 16:4% CCR:RHA from 5.6 × 10^−7^ m^2^/s with 20:0% CCR:RHA; which is also lower than 6.8 × 10^−7^ m^2^/s for 8% cement stabilized CEBs (Table 2). Table 1 and Table 2 further present the average values of the thermal conductivity and diffusivity along with their CV which recorded similar variations for all CEBs (CV < 10). Figure 4c similarly shows the correlation between the thermal conductivity and diffusivity with the bulk density for CEBs stabilized with CCR. Figure 4c,d also present the possibility to minimize the values (in the circles) of the thermal conductivity and diffusivity with respect to the bulk density of CEBs.

In the present study, the thermal conductivity of CEBs stabilized with the CCR and CCR:RHA reached the values lower or comparable to the values reported in the literature for unstabilized CEBs of similar bulk density. Similar observation can be made on the thermal diffusivity [21,26]. Moreover, it was reported that the thermal conductivity and diffusivity of unstabilized CEBs decreased with the bulk density [26]. This may suggest that the chemical stabilization is not the direct factor affecting the thermal properties of CEBs; rather the evolution of the resulting bulk density. Note that the stabilization with the CCR (0–25%) decreased the bulk density (1800–1480 kg/m^3^), increased the total porosity (35–45%), which decreased the thermal conductivity and diffusivity.

This can partly explain why the values of the thermal conductivity and diffusivity, in the present study, are lower than the values reported in the literature, particularly given that the air contained in the pores has lower values (0.03 W/m∙K and 2.2 × 10^−5^ m^2^/s, respectively). Therefore, it can be deduced that the type of earthen materials and stabilizer possibly influence the thermal properties through the evolution of the bulk density/porosity resulting from the chemical stabilization and compression of CEBs. Another explanation of this difference can be related to the variability in equipment and methods of measurement. This points out that stabilized CEBs can be engineered during production to control their thermal properties, without compromising the mechanical performances.

#### 3.2.3. Thermal Efficiency

The thermal efficiency was assessed on the basis of the evolution of thermal penetration depth in the CEBs. This is a parameter relating the thermal properties of materials for a period of 24 h. The depth evolved in the range of 0.13 to 0.11 m for CEBs stabilized with 0–25% CCR (Table 1). It reached the minimum value of 0.106 m for CEBs stabilized with 14:6% CCR:RHA from 0.124 m for CEBs stabilized with 20:0% CCR:RHA, compared to 0.136 m for CEBs stabilized with 8% cement (Table 2).

On the one hand, this shows that the heat flux may not cross through the total thickness (0.14 m) of the CEBs after the period of 24 h. On the other hand, this parameter can be used as basis to design the appropriate size of the wall masonry (thickness of CEBs) which would have efficient thermal performance. For comparison, the thermal penetration depth was deduced from the values of thermal diffusivity reported in the literature. It suggested that the thickness should be in the range of 0.09–0.11 m or 0.12 m for CEBs stabilized with 0–10% geopolymer or 8% cement [21] and 0.14–0.19 m for unstabilized CEBs with bulk density of 1600–2200 kg/m^3^ [26].

In fact, a simulation study [32] evidenced the thermal and energy efficiency of a building (wall) constructed using CCR stabilized CEBs compared to hollow cement blocks (HCB) based building. The CCR-CEBs-building allows to achieve lesser warm discomfort (400 h lesser) than HCB-building, only using natural ventilation systems. Moreover, if an air conditioner is used to keep the average temperature at 28 °C in both buildings, the CCR-CEBs-building allows to save 535 USD per year on electricity consumption for operation. Furthermore, the CEBs in the present study (stabilized with by-products) can be regarded as thermally efficient given that they have thermal conductivity and specific capacity which are respectively lower than 1 W/m∙K and higher than 920 J/kg∙K, with respect to the Brazilian standard for blocks and tiles [33].

#### 3.2.4. Effect of Water Content on the Thermal Properties

**Figure 5:**
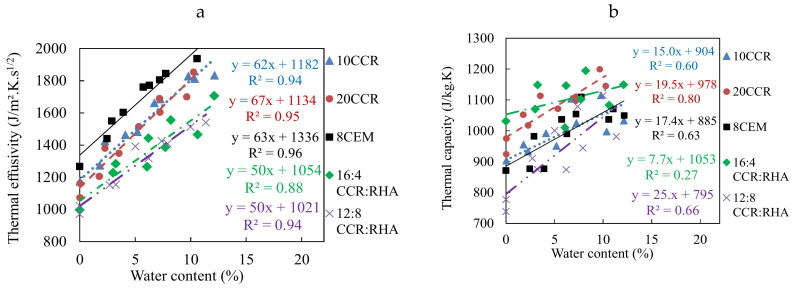
Evolution of (**a**) the thermal effusivity and (**b**) capacity of CEBs with water content.

**Figure 6:**
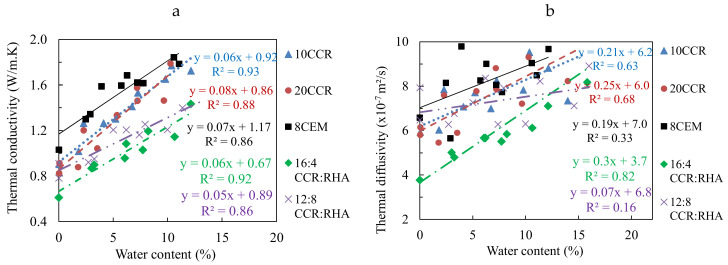
Evolution of the thermal (**a**) conductivity and (**b**) diffusivity of CEBs with water content.

The thermal properties of CEBs stabilized with CCR and CCR:RHA increased with respect to the content of liquid water (Figure 5 and Figure 6). The evolution of some thermal properties (τ) can tentatively be assimilated to linear correlations, τ = a × wc + b, increasing at different rates (a) with respect to the water content (wc) from the initial values in dry state (b). Although not reported, the experimental results showed that this correlation is no more valuable beyond 15% water content. Similar observation was previously made by Meukam [34].

The thermal effusivity increased in the range of 1000 to 2000 J/m^2^·K·s^1/2^, at rate, a, of 50–70 J/(m^2^∙K∙s^1/2^ %) for water content in the range of 0–10% (Figure 5a). The thermal capacity evolved in the range of 800 to 1200 J/kg∙K, at rate of 15–25 J/(kg∙K %), except 7.7 J/(kg∙K %) with 16:4% CCR:RHA (Figure 5b). The thermal conductivity increased in the range of 0.6 to 2 W/m∙K, at rate of 0.05–0.08 W/(m∙K %) (Figure 6a). The thermal diffusivity evolved in the range of 4 × 10^−7^ to 10 × 10^−7^ m^2^/s, without showing reasonable correlations with the water content (Figure 6b). This increase can be related to the higher values of thermal effusivity (1588 J/m^2^∙K∙s^1/2^), capacity (4180 J/kg∙K) and conductivity (0.6 W/m∙K) of liquid water compared to the values for air (6 J/m^2^∙K∙s^1/2^, 1004 J/kg∙K, 0.03 W/m∙K, respectively) in the porosity of CEBs. The water acts as bridge for thermal conduction and/or through diffusion. This suggests that the thermal properties of CEBs can be estimated at various water content knowing their thermal properties in dry state.

The thermal conductivity of CEBs stabilized with by-product binders evolved with water content similarly to few studies described in the literature; they showed that the rate of increase is around 0.06 W/(m∙K∙%) [2,34,35]. Bogas et al. [27] also claimed that the thermal conductivity of water saturated CEBs (water content of 13–16%) was 2–2.4 times higher than that of dry CEBs. In the present study, the thermal conductivity reached values two times higher than that of dry CEBs before saturation (water content of <15%). This suggests that the more the CEBs absorb water, the higher the thermal conductivity increases. Otherwise, no other studies, to the best knowledge of the authors, reported on the influence of water content on the thermal properties of CEBs. Therefore, it is essential to assess the hygrothermal properties of CEB in order to better exploit their potential for hygrothermal regulation in buildings, and especially in the hot climates with non-air conditioning systems [36].

#### 3.2.5. Sorption of Water Vapor

**Figure 7:**
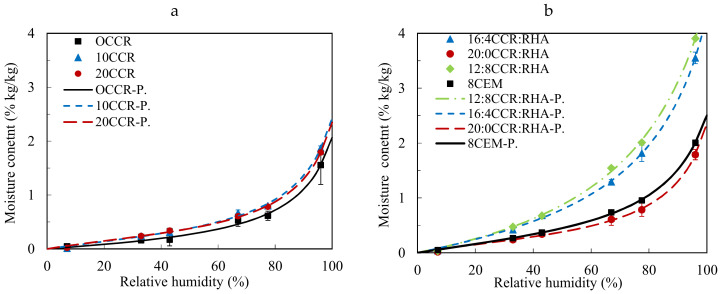
Sorption isotherms of CEBs stabilized with (**a**) CCR and (**b**) CCR:RHA: fitted with GAB model (P.), R^2^ > 0.99.

Following up the understanding of the hygrothermal performances of CEBs, Figure 7 presents the sorption behavior of CEBs stabilized with CCR and CCR:RHA. It shows that the equilibrium moisture content (EMC) adsorbed by CEBs increases with the relative humidity (Ψ: 7–95%) of the environment (20 ± 2 °C). The isotherms were fitted very well (R^2^ > 0.99) using the GAB (Guggenheim-Anderson-de Boer) model describing the evolution of the EMC with Ψ. The isotherms of all CEBs have similar shape, type III, according to the classification of BET (Brunauer–Emmet–Teller) models [37]. Some studies previously reported the isotherms of the same shape for CEBs [35,38]. Although, type II isotherm is usually the most common for porous building materials [39], including some earthen materials [1]. The type III isotherm is reported for the rare cases of nonporous adsorbents with very small interactions between the adsorbent and adsorbed medium [37].

The type III isotherm presents only one point of inflection at higher Ψ (about 80% in the present study) which is characteristic of the beginning of the main adsorption mechanism (capillary condensation), with limited adsorption at lower Ψ [3,37,38,40]. This suggests that the CEBs in the present study adsorbed water vapor molecules mainly by capillary condensation process in the micropore and mesopore. The electrostatic adsorption by van der Waals forces or hydrogen bonds on the surface of the pore does not seem to be the major physical process [37,38]. It can be related to the low activity/interaction of the kaolinite, the main clay mineral in the earthen material, with water molecules.

The isotherms of the CEBs stabilized with 10 and 20% CCR are similar to that of unstabilized CEBs (0% CCR). They reached the total EMC in the range of 1.6–1.8 %, i.e., the mass percentage of water vapor adsorbed per mass of the CEBs sample (Figure 7a). The isotherms of CEBs stabilized with CCR:RHA recorded the highest EMC in the range of 3.5–3.9 %, marking a clear difference with respect to the CEBs stabilized with CCR alone or cement (Figure 7b).

Different studies previously claimed that the stabilization, with cement and/or lime, reduces the moisture sorption/buffering capacity of earthen materials [1,3,41]. By contrast, it is not the case in the present study where the EMC of CCR-stabilized CEBs showed same evolution as the unstabilized CEBs. Similarly, the substitution of the CCR by the RHA resulted in remarkable increase of EMC of CEBs. The reduction of the moisture sorption after stabilization was previous related to the densification of earthen matrix by hydrated cementitious products which blocked the pore spaces and prevented the air from circulating [1]. In the present study, it was not the case for the CEBs stabilized with CCR or CCR:RHA which have even higher porosity, thus better moisture sorption capacity than the unstabilized CEBs.

The type of clay is another factor influencing the moisture sorption of earthen materials. The materials containing active clay (high specific surface area), such as montmorillonite, would have higher sorption capacity than non-active clay such as kaolinite [3,23,35,42]. Liuzzi et al. [43] reported high isothermal vapor sorption capacity for lime stabilized bentonite composite compared to other clay-based composite. Nevertheless, the sorption behavior of CEBs in the present study are comparable to that in the literature for kaolinite rich-earthen materials [41].

It can be concluded that the stabilization of CEBs using by-product binders not only improves the mechanical performances but also the hygrothermal performances. It increases the compressive strength and decreases the bulk density (increases the porosity), inducing the decrease of thermal conductivity and diffusivity and increase of moisture sorption capacity. Similarly to the thermal properties, it can be assumed that the sorption of CEBs is indirectly affected by the stabilization through the textural modification (density, porosity). This again gives possibility to control the sorption capacity through the production process. Nevertheless, increasing the total porosity of CEBs may affect the durability. Therefore, further studies should assess the effect on the long term performances such as resistance to water absorption, abrasion or erodability.

## 4. Conclusions

This paper investigated the engineering properties of CEBs resulting from the stabilization with by-product binders. The CCR and RHA were responsible for the improvement the physico-mechanical and hygrothermal properties of stabilized CEBs, mainly with at least 10% CCR or 18:2 to 16:4% CCR:RHA, such that:

The bulk density of CEB stabilized with CCR and CCR:RHA evolved in the range of 1710 to 1550 kg/m^3^. This is lower than 1801 kg/m^3^ for unstabilized CEBs and 1781 kg/m^3^ for 8% cement-stabilized CEBs. It is accompanied by the increase of the total porosity in the range of 35 to 45%.

The compressive of CCR-stabilized CEBs surpassed 4 and 2 MPa with 10% CCR respectively in dry and wet conditions, as required for the constructions of two-storey buildings. The partial substitution of 20% CCR by RHA (CCR:RHA) further improved the compressive strength beyond 6 MPa with 16:4% CCR:RHA, required for applications in three-storey building. However, the CEBs should not be exposed to wet environment, using surface coating or architectural protection systems, as the wet compressive strength failed to reach 3 MPa.

The structural efficiency of the stabilized CEBs was evidenced by the decrease of the bulk density and the increase of the compressive strength. The coefficient of structural efficiency (CSE) of CEBs increased more than three times due to the stabilization with the CCR. This suggests that the more the CCR content, the better the bearing capacity of stabilized CEBs. The CSE further reached the maximum with 16:4% CCR:RHA; it is not only the optimum design regarding the compressive strength, but also the bearing capacity, which is even better than cement stabilized CEBs. This highlights a positive effect of the decrease of bulk density on the load carrying capacity of CEBs.

The thermal efficiency of stabilized CEBs is shown by the decrease of thermal effusivity and increase of thermal capacity. This resulted in the decrease of thermal conductivity from 1.02 W/m∙K for unstabilized CEBs to 0.69 W/m∙K or 0.64 W/m∙K, respectively, for CEBs stabilized with 25% CCR or 16:4% CCR:RHA. It is also accompanied by the decrease of thermal diffusivity and thermal penetration depth.

Furthermore, the thermal property (τ) of stabilized CEBs increased with water content (wc) and can be estimated as τ = a × wc + b, knowing their values in dry state (b). The rate (a) is estimated around 50–70 J/(m^2^ K s^1/2^∙%), 15–25 J/(kg∙K∙%), 0.05–0.08 W/(m∙K∙%), respectively, for the thermal effusivity, capacity and conductivity. Moreover, the stabilization of CEBs with the by-products improved the sorption capacity. The total equilibrium moisture content of 1.6–1.8% was adsorbed by the unstabilized and CCR-stabilized CEBs and 3.5–3.9% by the CCR:RHA stabilized CEBs, which was higher than cement-CEBs. However, further investigation should be carried to assess the effect of stabilization with these by-products on the key indicators of the durability of CEBs.

## Figures and Tables

**Table 1:** Summary of the physico-mechanical and thermal properties of CEBs stabilized with CCR.

Binder Content (%)		Bulk Density (Kg/m^3^)		Total Porosity, TP (%)		Compressive Strength (MPa)				CWS *	CSE * (J/kg)	Thermal										
												Effusivity, E (J/m^2^ K·s^1/2^)		Capacity, Cp (J/kg∙K)		Conductivity, λ (W/m∙K)		Diffusivity, a (m^2^/s)		Penetration Depth, δp (m)		
						Dry		Saturated														
CCR	0	1801	1	35	1	1.1	3	ND *	ND *	ND *	609	1291	3	899	1	1.02	6	6.3 × 10^−7^	10	0.132	5	
	5	1711	1	38	1	3	3	1.6	6	0.5	1767	1152	1	922	1	0.89	5	5.6 × 10^−7^	6	0.121	1	
	10	1689	1	38	2	4.3	10	2.7	6	0.6	2528	1159	4	916	1	0.84	1	5.4 × 10^−7^	1	0.122	0	
	15	1573	0	42	1	4.6	5	1.9	5	0.4	2901	1084	3	957	0	0.79	4	5.2 × 10^−7^	2	0.119	3	
	20	1522	1	44	1	4.4	7	1.8	7	0.4	2902	1107	1	966	1	0.83	2	5.6 × 10^−7^	1	0.124	1	
	25	1477	1	45	1	4.5	3	1.7	4	0.4	3053	1010	4	997	1	0.69	6	4.7E × 10^−7^	2	0.114	1	

* CWS: coefficient of water strength, CSE: coefficient of structural efficiency, coefficient of variation (%) = the percentage ratio between the values of standard deviation and average, ND: not determined.

**Table 2:** Summary of the physico-mechanical and thermal properties of CEBs stabilized with CCR:RHA: average values and coefficients of variations *.

Binder Content (%)		Bulk Density (Kg/m^3^)		Total Porosity, TP (%)		Compressive Strength (MPa)				CWS *	CSE * (J/kg)	Thermal									
						Dry		Saturated				Effusivity, E (J/m^2^·K·s^1/2^)		Capacity, Cp (J/kg∙K)		Conductivity, λ (W/m∙K)		Diffusivity, a (m^2^/s)		Penetration Depth, δp (m)	
CCR:RHA	20:0	1522	1	44	1	4.4	7	1.8	7	0.4	2902	1108	1	966	1	0.83	2	5.6 × 10^−7^	1	0.124	1
	18:2	1578	1	41	0	6.4	4	2.4	4	0.4	4045	1042	1	942	1	0.73	1	4.9 × 10^−7^	1	0.116	0
	16:4	1575	1	40	1	7	3	2.7	6	0.4	4462	974	3	939	2	0.64	8	4.1 × 10^−7^	0	0.106	0
	14:6	1577	1	40	1	6.8	6	2.3	4	0.3	4293	1009	1	933	2	0.69	0	4.7 × 10^−7^	2	0.114	1
	12:8	1555	2	39	0	6	5	1.9	9	0.3	3827	1081	1	880	1	0.86	3	6.3 × 10^−7^	4	0.131	2
CEM *	8	1781	8	37	1	6.2	4	3.1	3	0.5	3547	1231	3	844	6	1.01	7	6.8 × 10^−7^	10	0.136	5

* CWS: coefficient of water strength, CSE: coefficient of structural efficiency, coefficient of variation (%) = the percentage ratio between the values of standard deviation and average, CEM: Cement.
